# A framework for TRIM21-mediated protein depletion in early mouse embryos: recapitulation of *Tead4* null phenotype over three days

**DOI:** 10.1186/s12864-019-6106-2

**Published:** 2019-10-21

**Authors:** Steffen Israel, Ellen Casser, Hannes C.A. Drexler, Georg Fuellen, Michele Boiani

**Affiliations:** 1 Max Planck Institute for Molecular Biomedicine, Roentgenstrasse 20, 48149 Muenster, Germany; 2 Rostock University Medical Center, Institute for Biostatistics and Informatics in Medicine and Aging Research (IBIMA), Ernst-Heydemann-Strasse 8, 18057 Rostock, Germany

**Keywords:** Proteome, TEAD4, Oocyte, Preimplantation embryo, TRIM21, Mouse, Trophectoderm

## Abstract

**Background:**

While DNA and RNA methods are routine to disrupt the expression of specific genes, complete understanding of developmental processes requires also protein methods, because: oocytes and early embryos accumulate proteins and these are not directly affected by DNA and RNA methods. When proteins in the oocyte encounter a specific antibody and the **TRI**partite **M**otiv-containing **21** (TRIM21) ubiquitin-protein ligase, they can be committed to degradation in the proteasome, producing a transient functional knock-out that reveals the role of the protein. However, there are doubts about whether this targeted proteolysis could be successfully used to study mammalian development, because duration of the transient effect is unknown, and also because amounts of reagents delivered must be adequate in relation to the amount of target protein, which is unknown, too.

**Results:**

We show that the mouse egg contains up to 1E-02 picomoles/protein, as estimated by mass spectrometry using the intensity-based absolute quantification (iBAQ) algorithm. However, the egg can only accommodate ≈1E-04 picomoles of antibody or TRIM21 without incurring toxic effects. Within this framework, we demonstrate that TRIM21-mediated protein depletion efficiently disrupts the embryonic process of trophectoderm formation, which critically depends on the *TEA domain family member 4* (*Tead4*) gene. TEAD4 depletion starting at the 1-cell stage lasts for 3 days prior to a return of gene and protein expression to baseline. This time period is long enough to result in a phenotype entirely consistent with that of the published null mutation and RNA interference studies: significant underexpression of trophectodermal genes *Cdx2* and *Gata3* and strongly impaired ability of embryos to cavitate and implant in the uterus. Omics data are available via ProteomeXchange (PXD012613) and GEO (GSE124844).

**Conclusions:**

TRIM21-mediated protein depletion can be an effective means to disrupt gene function in mouse development, provided the target gene is chosen carefully and the method is tuned accurately. The knowledge gathered in this study provides the basic know-how (prerequisites, requirements, limitations) to expedite the protein depletion of other genes besides *Tead4*.

## Background

Classic DNA techniques of gene ablation (constitutive ‘knockout’) eliminate gene function ubiquitously in 25% of the mouse embryos produced after the intercross of heterozygous founders. In this way, for example, the requirement of the transcription factor TEA domain family member 4 (TEAD4) has been demonstrated for mouse preimplantation development [1, 2]: Intercross of *Tead4* +/− parents produced no *Tead4* −/− offspring, because null embryos died at pre-implantation stages without forming a blastocyst cavity encased in a functional trophectoderm. In other gene mutants, e.g. *Pou5f1 (Oct4),* null embryos were able to form blastocysts only to die shortly after implantation [3]. These phenotypes were also reproduced by inhibiting the mRNA via RNA interference or morpholino, as shown for *Tead4* itself [4, 5] and its target gene *Cdx2* [6].

However, protein methods are indispensable for a complete understanding of developmental processes, because oocytes and early embryos accumulate proteins and these are not directly affected by the above DNA and RNA methods. Specifically, proteins can outlive the locus deletion (in knockout models) or the inhibition of cognate mRNA (in siRNA/morpholino experiments). Apart from exceptional cases of proteins with half-lives ranging from months to years [7], some embryonic proteins remain there for days after the cognate mRNA has been degraded (e.g. NLRP2 and members of the subcortical maternal complex, SCMC [8, 9]). These considerations fuel speculation that some null mutant phenotypes might be only partly revealed by DNA and RNA methods. Therefore it is desirable to eliminate the proteins directly. One possibility is to microinject, into the oocyte, IgG antibodies either alone [10–18] or in combination with a suitable E3 ubiquitin-protein ligase, such as TRIM21, that binds IgG [19, 20]. Antibodies alone mask the target proteins at the catalytic or interaction sites, but the target proteins are not eliminated. By adding TRIM21, an antibody-target-TRIM21 ternary complex is formed that is degraded in the proteasome [21], thus producing a functional ‘knockout’. In mouse oocytes the TRIM21-mediated protein depletion has been demonstrated on two endogenous proteins and on microinjected green fluorescent protein (GFP), which all rapidly disappeared from oocytes for at least 60 min (t ½ 9–16 min) [21]. In embryos, depletion has been conducted in Zebrafish via microinjection in the egg yolk, producing phenotypes in the embryos [22].

In order to be feasible in mammalian development and be applicable to more questions to come, such as the role of maternal protein deposits in oocytes, TRIM21-mediated protein depletion needs to fulfill basic operating criteria. The native range of protein amounts that TRIM21 is supposed to deplete has to be defined, for example, and the supplied amount of antibody must be maximized, in order to possibly last for e.g. 3–4 days of mouse preimplantation development. While ≈5000 proteins are detectable in MII mouse oocytes with current technology [23, 24], they have not been assigned yet with a unit of substance e.g. femto-, pico- or nanomoles. These target protein amounts must be matched, or exceeded, by the antibody supplied in a microinjected volume. While Zebrafish oocytes can be injected with 2000 picoliters containing 6,7E-03 picomoles antibody [25], mouse oocytes are smaller and injection volumes range from 1 to 20 picoliter [26]. Experimenters have traditionally been reluctant to inject more than 5–10 picoliters: protein injection in the mouse ooplasm was able to deliver 2E-05 picomoles in 7 picoliters, for example [27]. Yet mature mouse oocyte and zygotes should be able to accommodate for a volume expansion of approximately 100 picoliters, thanks to the large perivitelline space [28, 29], but this has not been demonstrated yet. In addition to these parameters, the antibody still might not bind all of the target protein, given the chemical equilibrium law. In fact, the binding properties of most antibodies are not well characterized [30].

Aim of the present study was to define an operating framework for TRIM21-mediated protein depletion in mouse development. To this end we chose the paradigm of trophectoderm formation in mouse embryos, which hinges on the transcription factor TEAD4. This choice offers key advantages, such as a well-characterized gene expression cascade [5, 31–34] and an easy-to-assess dichotomic phenotypic response (blastocyst formation yes/no [1, 2]). The knowledge gathered in our study illustrates prerequisites, requirements and limitations of TRIM21-mediated protein depletion in early mouse embryogenesis, and paves the way to study the function of protein deposits in mouse oocytes.

## Results

### Operating criterion no.1 for successful TRIM21-mediated protein depletion: known amount of target protein

In order to be feasible in mammalian development for the study of gene functions, TRIM21-mediated protein depletion needs to be adapted to a cell type that is specialized to store proteins in large amounts and release them over days: the fertilized oocyte, or zygote. Therefore, our first consideration was to define the native range of protein amounts that TRIM21 is supposed to deplete, so as not to operate blindly. To date, the molar abundance of proteins present in mouse oocytes or early embryos is essentially unknown. While there are deep quantitative studies [23, 24], these provide relative comparisons (e.g. the amount of protein P in sample X is larger than in sample Y), but these quantities are not scaled as moles. Using cell lines and mass spectrometry (MS) it has been demonstrated that a protein’s abundance as a fraction of the total protein is reflected by the proportion of its MS intensity signal to the total MS intensity. The intensity-based absolute quantification (iBAQ) algorithm divides the sum of all precursor-peptide intensities by the number of theoretically observable peptides for the corresponding protein [35, 36]. iBAQ values are approximately proportional to the number of moles of protein present and thus iBAQ_i_/Σ_j_iBAQ_j_ (adimensional) is the relative molar amount of protein ‘i’ among all proteins ‘j’, called relative iBAQ, briefly riBAQ [37].

**Fig. 1:**
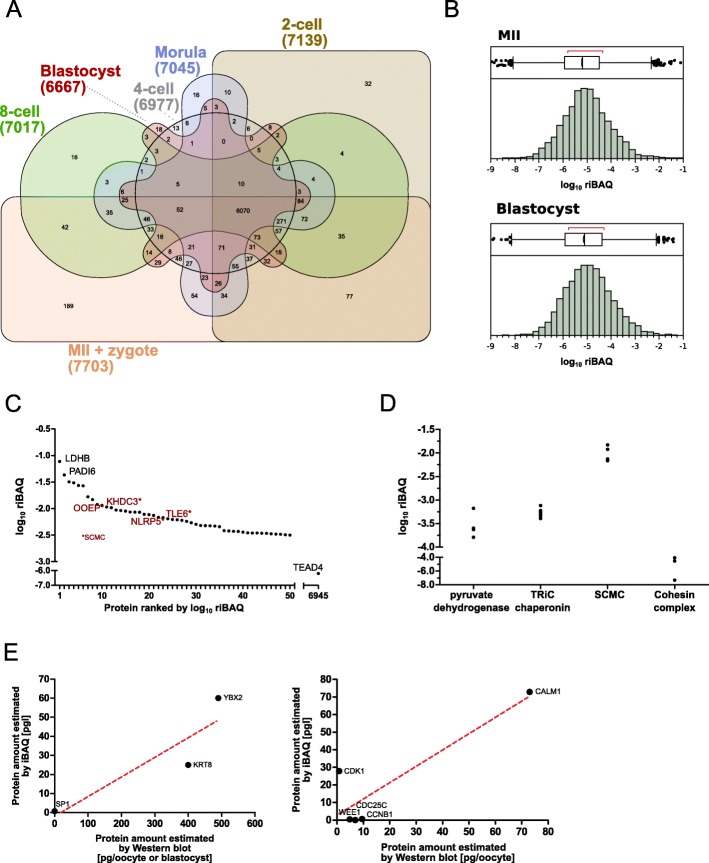
**a.** Venn diagram overview [38] of the number of protein identities detected in oocytes and preimplantation stages of the mouse following LC-MS/MS. **b.** Distribution of individual protein abundances (riBAQ) in oocytes and blastocysts. **c.** Survey of the top 50 most abundant proteins detected in oocytes, plus TEAD4. **d.** Demonstration that the subunits of four known macromolecular complexes are quantified at similar riBAQ values within each complex. **e.** Tentative picogram amounts of protein p predicted with the formula *M*_*p*_ = *riBAQ*_*p*_ × *MW*_*p*_ are consistent with those found in historical immunoblotting data Abbreviations: MS, mass spectrometry; WB, western blotting; MII, metaphase II oocyte

To obtain riBAQ values for mouse preimplantation stages we repurposed and reprocessed a large series of previously generated MS datasets (see Methods). These datasets covered seven stages (metaphase II (MII) oocyte, pronuclear stage 2 (PN2) zygote, 2-cell, 4-cell, 8-cell, ≈16-cell or morula, and ≥ 32-cell or blastocyst) in four replicates per stage except *n* = 5 for oocytes. Combined, these data assembled to 8095 protein groups (MII, 7676; zygote, 7362; 2-cell, 7139; 4-cell, 6977; 8-cell, 7017; morula, 7045; blastocyst, 6667) (Fig. 1a). The MS proteomics data have been deposited to the ProteomeXchange Consortium via the PRIDE partner repository [39, 40] (see Methods). A summary of the processed riBAQ values is provided as Additional file 1: Table S1. In oocytes, these proteins span 8 orders of magnitude of riBAQ values (Fig. 1b, top), ranging from 4.0 × 10^− 9^ to 7.7 × 10^− 2^ with a median of 9.0 × 10^− 6^. For convenience we will adopt the scientific notation: 4.0 E-09, 7.7 E-02, 9.0 E-06. Strikingly, 50 proteins alone (≈0.6% of 8095) accounted for 50% of the sum of all iBAQ values in oocytes (Fig. 1c). These highly abundant proteins include LDHB, PADI6, and all four core members of the SCMC, which have been described as highly abundant also in previous proteomic studies [41]. The intermediate riBAQ range of E-07 to E-05 is populated by proteins which include transcription factors associated with preimplantation development, such as OCT4 and TEAD4. While OCT4 is detected in all (29 of 29) replicates (65th abundance percentile in oocytes), TEAD4 is detected in 5 of 29 replicates (1 of 5 replicates for oocytes; 8th abundance percentile). Clearly, the view of transcription factors as little abundant proteins is a generalization. Since the onset of embryonic *Tead4* gene expression is at the 2-cell stage [2], our case refers to a preexisting (albeit very minute!) protein deposit in oocytes, a deposit that would be immune to DNA and RNA methods, analogous to OCT4, which however is more abundant. Compared to the distribution of riBAQ values for oocytes, similar distributions are obtained also for the developmental stages, as shown exemplarily for blastocyst (Fig. 1b, bottom). This quantitative knowledge will be instrumental to scale the amounts of TRIM21 and antibody required for target protein depletion.

How faithfully does the riBAQ algorithm report the mole fraction of each protein in case of mouse oocytes and early mouse embryos? And can a unit of substance (e.g. picomole) be tentatively assigned to the riBAQ values? To answer these questions we performed two assays, as follows.

First, we reasoned that macromolecular complexes offer an option for probing molar fractions. Many protein complexes are well characterized in terms of their composition and stoichiometry, with subunits expressed at equimolar levels, as deduced from co-immunoprecipitation experiments. Two such complexes present in all cells are the pyruvate dehydrogenase complex and the TRiC chaperonin, already used in previous MS studies to validate protein amounts [42]. Specific to oocytes, two such complexes are the highly abundant SCMC [43] and the less abundant cohesin complex [44]. In our dataset, not only did we find all expected members of the ubiquitous complexes, but also very similar amounts of the respective subunits at riBAQ level of less than E-03 (pyruvate dehydrogenase, TRiC chaperonin). Of the SCMC we found all four subunits at riBAQ level of less than E-1.5 (Fig. 1c, d). Of the cohesin complex we found 3 of 4 constitutive members at a riBAQ level less than E-04, with riBAQ of SMC1A similar to riBAQ of SMC3 and both riBAQs higher than that of STAG3, consistent with the stoichiometry of SCMC (Fig. 1d). Cohesin complex subunit REC8 was not found.

Second, we reasoned that if the riBAQ of protein *p* is proportional to its molar fraction MF_*p*_,

1 $$ {MF}_p\propto {riBAQ}_p, $$

then multiplying the riBAQ value of a protein by its molecular weight (MW) in Daltons (g/mol, or picogram/picomole) would yield the protein mass Mp

2 $$ {M}_p={riBAQ}_p\times {MW}_p $$

and finally, summing up these products M_*i*_ for all the *n* proteins detected in an oocyte sample, should return a mass M_*O*_

3 $$ {M}_O={\sum}_1^n{M}_p $$

that compares well to the known total protein mass of the mouse oocyte, which is 2.4E+ 04 ± 0.5E+ 04 picograms [45–50]. Indeed the obtained M_O_ value of 4.4E+ 04 not only is in the same order of magnitude but in fact it is close to the average value from literature [45–50]. Even though the current proteome is incomplete because there are proteins that went undetected in our MS analysis (e.g. REC8), these are likely to be the least abundant proteins, whose share M_i_ of the total M_O_ is negligible. Therefore, the riBAQ values are proportional to an entity that carries picomole as a unit of substance. Tentative picogram amounts predicted with formula (2) are consistent with those found in historical immunoblotting data (YBX2 [51], SP1 [52] and KRT8 [18] in MII oocytes and in blastocysts, which can be matched directly to our samples; CALM1 [53], WEE1, CDK1, CCNB1, and CDC25C [54] in fully grown, germinal vesicle oocytes, which can be related by approximation to our samples of MII oocytes; Fig. 1e).

In sum, the riBAQ values reflect the protein abundance in situ and are proportional - albeit not identical - to the picomoles of protein present in oocytes and preimplantation embryos. As such, riBAQ values can guide the supply of specific antibody (e.g. ensuring that the antibody is in excess of the target protein) and can help interpret the results of TRIM21-mediated protein depletion.

### Operating criterion no.2 for successful TRIM21-mediated protein depletion: duration and selectivity of effect

**Fig. 2:**
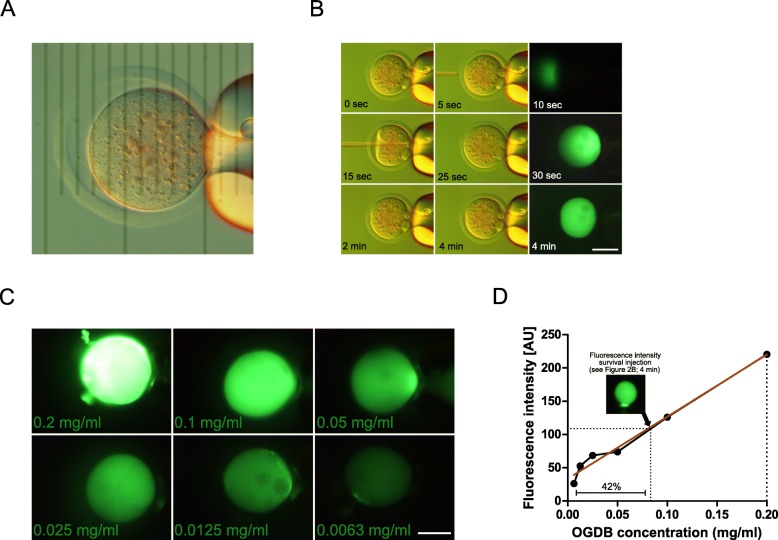
**a.** Picture of a metaphase II mouse oocyte with the picture of a micrometre grid (10-μm intervals) superimposed. **b.** Zygotes were filmed during injection with a gentle flow of concentrated suspension (0.2 mg/mL), but only up to the filling of the perivitelline space with an injected volume ‘x’; selected frames were extracted from movie at indicated time points. Note that the perivitelline space is completely filled at 25 s, but recovers partly after 2 min and completely after 4 min. **c.** Serial doubling dilutions of a standard of green-fluorescent dextran beads (Oregon Green dextran beads, OGDB). Zygotes were blown up with a maximum flow of OGDB suspension applied continuously for 30 s, for each of six concentrations (0.2 mg/mL halved by serial dilutions down to 0.00625 mg/mL), causing the zona to be evacuated and the cytoplasm to be completely replaced by OGDB. The green fluorescence intensity was recorded for each dilution with the same excitation and time exposure (**d**)**.** Dilution factor: A zygote injected as in (B), shown in the small inset photo, had a fluorescence intensity corresponding to 42% (1/2.4) of the fluorescence of the standard alone. These data allow one to solve a simple equation for the injected volume ‘x’: dilution factor = 2.4 = (x + 220 pl) / x = 157 pl. Size bar, 50 μm. AU, arbitrary units of fluorescence intensity

Our next consideration was how to maximize the volume and the concentration of reagents (TRIM21 and antibody) for microinjection in the zygote. This is crucial, because the antibody does not regenerate itself while the target protein may have a turnover. A similar argument applies to TRIM21. Thus, it is not granted that effects of TRIM21-mediated protein depletion persist for enough time to phenocopy the loss of gene function in embryos. Consequently, we adapted our technique of blunt-tip, piezo-driven needle microinjection, which has been used for many years in our laboratory to perform nuclear transplantation into mouse oocytes. The average radius (r) of the ooplasm (37–38 μm) and the average radius of the inner side of the zona pellucida (42–43 μm) allow the calculation of their volumes as 4/3πr^3^ (Fig. 2a). Based on the radius and assuming spherical shape, we reasoned that it should be possible to inject ≈ 100 picoliters (inner volume of the zona pellucida, 320 picoliters, minus the volume of the ooplast, 220 picoliters) before the oolemma is pressed against the zona pellucida and the perivitelline space is filled. Upon microinjection, volume expansion is reversible in zygotes, which recover within 4 min in most cases (88 ± 10%, *N* > 1000; Fig. 2b), in contrast to MII oocytes that lyse much more often (35 ± 2% loss). We confirmed the estimate of 100 picoliters, by studying the behavior of a fluorescent stock solution (Oregon Green dextran beads, OGDB) injected in the zygotic ooplasm (Fig. 2c). Briefly, when a concentrated volume of OGDB is transferred into the recipient volume of a zygote, dilution occurs and the fold-change in fluorescence corresponds to the dilution factor, which in turn allows to calculate the injected volume using a calibration curve (Fig. 2d). This calculation leads to an estimate of 157 picoliters, which is even larger than 100 picoliters, probably because the zona is elastic and it can swell during microinjection. Regardless, these numbers corroborate that the volume we are microinjecting lies in the vicinity of 100 picoliters.

**Fig. 3:**
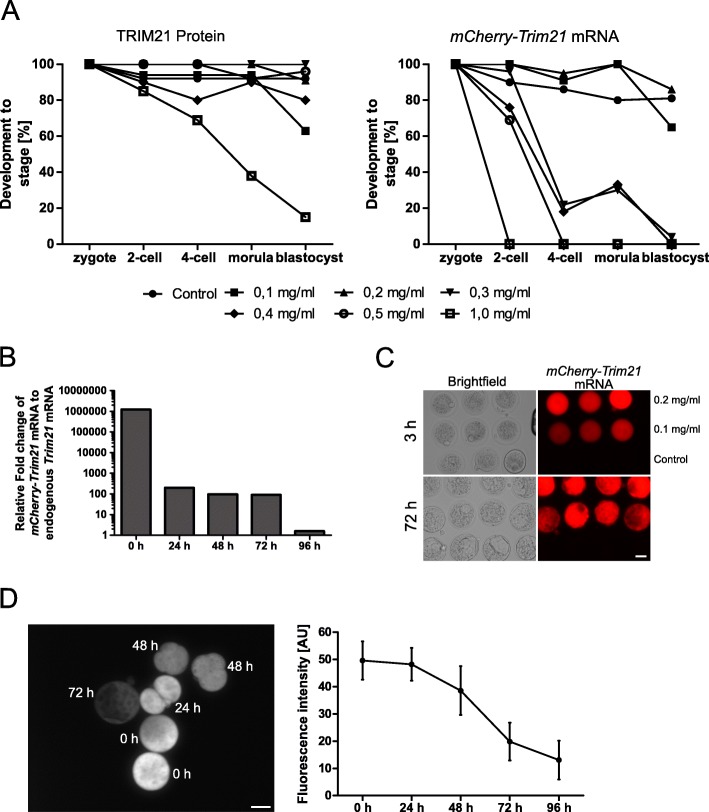
**a**. Dose-effect curves in zygotes injected with various concentrations of TRIM21 protein or *mCherry-Trim21* mRNA (mg/mL) in a volume of ≈ 100 picoliters. *N* = 43 zygotes were inspected for each mRNA concentration, *N* = 22 zygotes for each protein concentration. **b** Q-PCR for *Trim21* sequence conducted at 24-h intervals after microinjection of *mCherry-Trim21* mRNA in the zygote. *N* = 5 embryo equivalents from a lysate of 20 embryos per stage. Height of bars indicates the excess amount of microinjected *Trim21* mRNA over the endogenous *Trim21* mRNA. **c** The fluorescent protein product of *mCherry-Trim21* mRNA is visible already 3 h after microinjection and accumulates in the blastocysts. **d** Stability of microinjected antibody in the absence of *mCherry-Trim21* mRNA, demonstrated via immunofluorescence against primary antibody in zygotes microinjected with anti-GFP antibody. Left, representative picture of immunofluorescence against microinjected anti-GFP; right, measured fluorescence intensity (*n* = 7 embryos per time point). Size bar, 50 μm. AU, arbitrary units of fluorescence intensity

Next we addressed the concentration of solute in these 100 picoliters, i.e. the picomoles of TRIM21 and antibody that the zygote can tolerate without incurring cellular intoxication. We drew a dose-response curve increasing the concentration of protein, which was supplied salt-free in just water. We observed that concentrations above 0.5 mg/mL TRIM21 protein (52 kDa; 50 picograms of TRIM21 in ≈ 100 picoliters corresponding to 9.6E-04 picomoles) were followed by cytoplasmic protrusions oozing through the injection hole made in the zona pellucida, delay of volume recovery, and a marked decline of blastocyst rates (Fig. 3a left). On the assumption that this is due to the osmotic shock of TRIM21 being supplied all at once as protein, we injected the coding mRNA for a more gradual build-up of TRIM21 product. We took advantage of *Trim21* coding mRNA fitted with the coding sequence of mouse Cherry peptide (*mCherry-Trim21*), which allows to visualize TRIM21 in the living cell via red fluorescence [21]. We observed that concentrations above 0.2 mg/mL mRNA (730 kDa; 20 picograms of TRIM21 in ≈ 100 picoliters corresponding to 2.7E-05 picomoles) caused a decline of the blastocyst rates (Fig. 3a right; see also next paragraph). Therefore, we set the two concentrations of 2.7E-05 picomoles mRNA and 9.6E-04 picomoles antibody (in 100 picoliters) as the upper limit, and we introduced a safety margin, working with the lower concentrations of 2.5E-05 picomoles mRNA and 6.7E-04 picomoles antibody throughout this study. These numbers may look cumbersome, but in fact they correspond to 0.18 mg/mL mRNA and 1.0 mg/mL antibody. Using these settings, the amount of exogenous *Trim21* mRNA proved to be available throughout 72 h of preimplantation development: it remained substantially above the endogenous *Trim21* mRNA level (Fig. 3b) and was also effectively translated (Fig. 3c). Likewise, the amount of antibody proved to be stable throughout 72 h of preimplantation development (Fig. 3d). Thus, our microinjection technique should be applicable to study gene phenotypes that become manifest during the first 3 days of preimplantation development. Assuming that the antibody and its target follow a 1:1 stoichiometry, it should be possible to deplete up to 6.7E-04 picomoles of target protein, which falls in the 98th percentile of the iBAQ distribution (Fig. 1b). TEAD4 and OCT4, for example, lie well below (8th and 65th percentile, respectively, of the riBAQ distribution in oocytes).

**Fig. 4:**
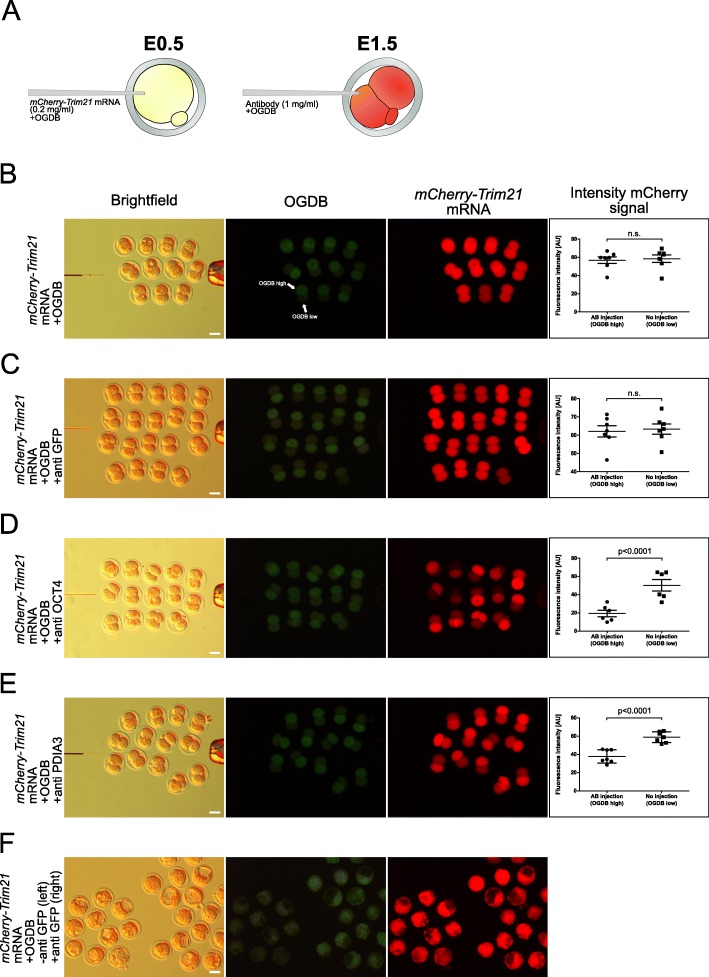
**a**. Experimental design of the selectivity test. All zygotes were injected with *mCherry-Trim21* mRNA and OGDB, cultured to the 2-cell stage and injected in one blastomere either with water (**b**), with anti-GFP antibody (**c**), with anti-OCT4 antibody (**d**) or with anti-PDIA3 antibody (**e**). **f.** The same as (**c**), followed up to the blastocyst stage (day E3.5). In the right-hand column, mCherry fluorescence was quantified using Image-J. Size bar, 50 μm. OGDB, Oregon Green dextran beads. Statistical significance tested with Student’s t test. n.s., not significant. AU, arbitrary units of fluorescence intensity

Our last consideration was to test whether the operation of TRIM21-mediated protein depletion through our microinjection protocol can, in principle, operate selectively in the mouse developmental environment and also preserve developmental ability. To stringently test the selectivity of the TRIM21 reaction, 2-cell embryos preloaded with *mCherry-Trim21* mRNA were injected with antibody into one blastomere, where the other one served as control. The injected blastomere can be discerned thanks to the brighter OGDB fluorescence (Fig. 4b-e). We relied for testing on three proteins that are known a priori to be present or absent in wild-type zygotes, such as PDIA3 and OCT4 (both present) and GFP (which is obviously absent in wild-type embryos). The estimated amounts (riBAQ) of PDIA3 and OCT4 in oocytes differ by a factor 240 (PDIA3 > OCT4; Additional file 1: Table S1). Within 4–5 h of the antibody injection, we could already see a robust decline of Cherry fluorescence for both anti-PDIA3 and anti-OCT4 (Fig. 4d, e), confirmed by signal intensity analysis (rightmost diagrams in Fig. 4d-e). The depletion of these proteins was selective, as shown by the fact that injection of anti-GFP did not result in any decrease of red fluorescence, neither after 4–5 h (Fig. 4c) nor after 3 days i.e. at the blastocyst stage (Fig. 4f). Blastocyst formation also demonstrates that microinjection per se does not grossly interfere with development.

### Demonstration that TRIM21-mediated protein depletion can be sustained for 3 days and phenocopies the genetic null phenotype of *Tead4*

**Fig. 5:**
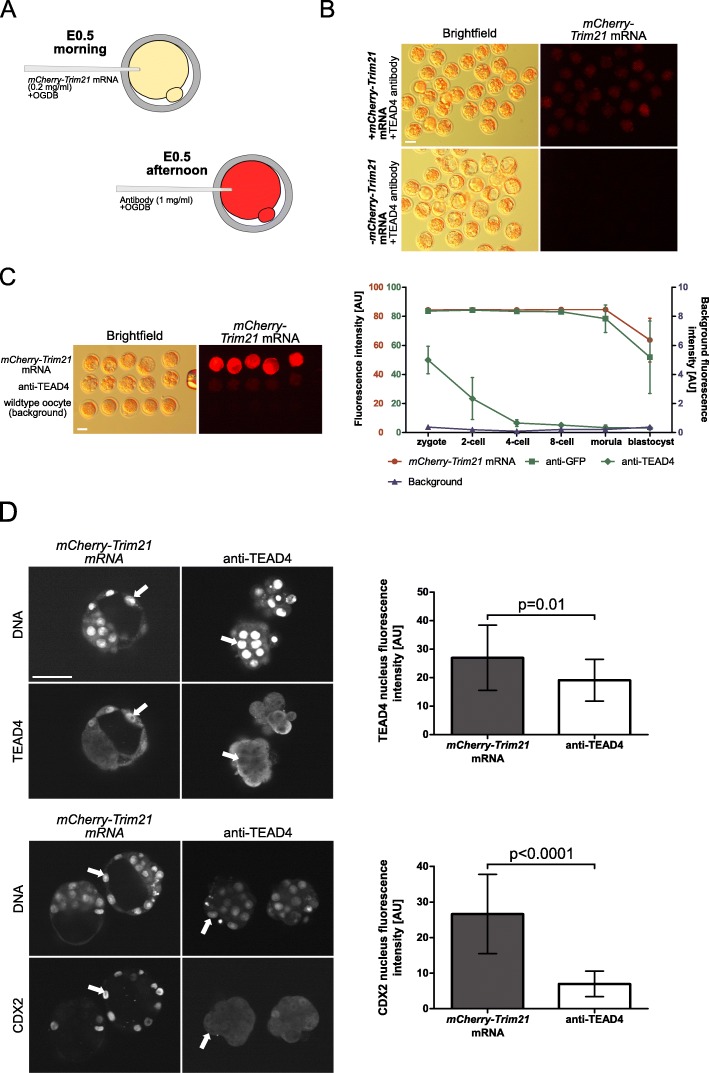
**a.** Experimental design for the investigation of developmental consequences of TRIM21-mediated protein depletion in zygotes. **b.** Representative images of blastocysts developed in KSOM (aa) after the microinjection of *mCherry-Trim21* mRNA and OGDB tracer with or without TEAD4 antibody. Effect of anti-TEAD4 was due to TRIM21-mediated depletion, as shown by the lack-of effect of TEAD4-antibody alone. **c.** Representative images of embryos with *mCherry-Trim21* mRNA and OGDB tracer; embryos with *mCherry-Trim21* mRNA, OGDB tracer and anti-TEAD4; non-injected oocytes with no fluorescence. Plot shows Cherry fluorescence intensities of zygotes and subsequent stages preloaded with *mCherry-Trim21* mRNA and OGDB tracer, and then injected with water (), anti-GFP antibody () or anti-TEAD4 antibody (), compared to the background fluorescence of non-manipulated cells (). *N* = 3 zygotes or embryos per stage per treatment. Note the secondary right axis used in plot to better discern the background fluorescence values. **d**. Representative immunofluorescent signals (largest cross section, nucleus fluorescence) of TEAD4 and CDX2 in TRIM21-only and TEAD4-depleted embryos at day E3.5 (*n* = 7 TEAD4-depleted and *n* = 8 TRIM21-only embryos for TEAD4 immunofluorescence; *n* = 11 TEAD-depleted and *n* = 8 TRIM21-only embryos for CDX2 immunofluorescence). DNA stained with YO-PRO-1. Arrows point at peripheral nuclei that are TEAD4- or CDX2-positive in controls but negative in TEAD4-depleted embryos. Size bar, 50 μm. OGDB, Oregon Green dextran beads. Error bars = standard deviations. Statistical significance tested with Student’s t test. AU, arbitrary units of fluorescence intensity

Under the conditions outlined above, effects of TRIM21-mediated TEAD4 protein depletion yielded the same phenotype (phenocopy) as the genetic *Tead4* mutant. When PN2 zygotes were injected with *mCherry-TRIM21* mRNA and ChIP-grade TEAD4 antibody [34] (Fig. 5a), embryos developed normally in vitro up to the 8-cell stage under culture conditions that mimic those in vivo (see Methods). However, compaction was abnormal and cavity formation was stunted under atmospheric O_2_ pressure (≈20%) after 3.5 days, during which time cavitation occurred in most of the control embryos injected with TEAD4 antibody alone (Fig. 5b). Thus, impairment was mediated by protein degradation via TRIM21, not by the antibody alone. Notably, blastocysts still had *mCherry-Trim21* mRNA and antibody left, as well as TRIM21 activity (Cherry fluorescence; Fig. 5c). This indicates that 1) reagents were not limiting and 2) depletion of the ternary complex and, thereby, of TEAD4 had occurred thus far.

**Fig. 6:**
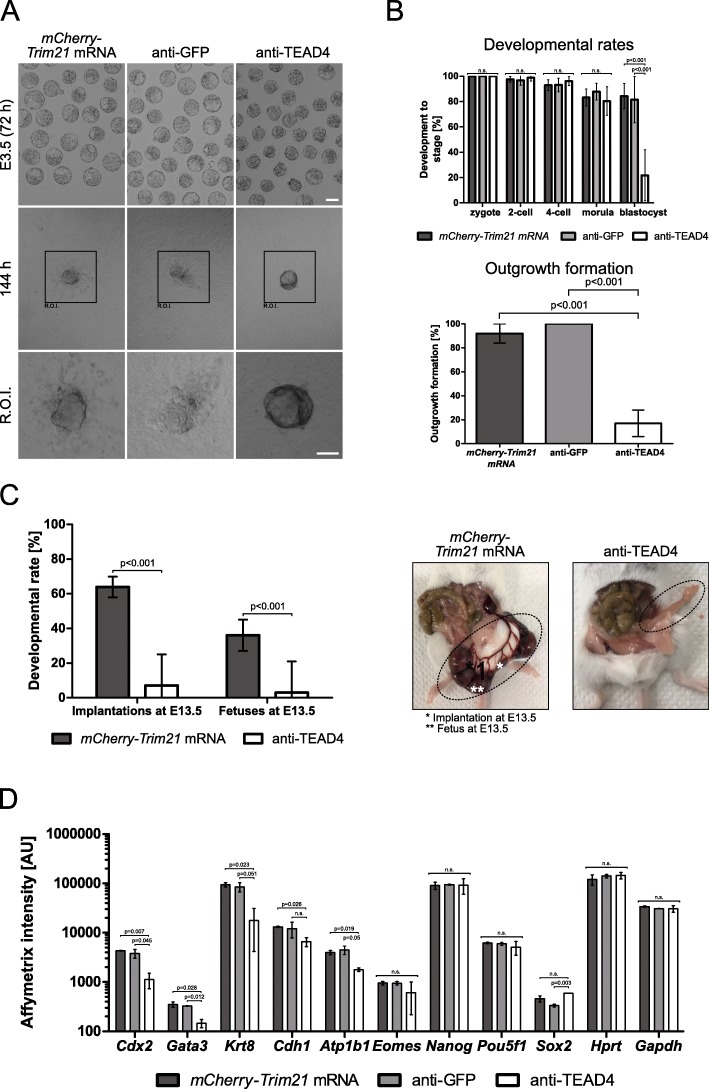
**a.** Morphologies of blastocysts and outgrowths with and without TEAD4 depletion. **b.** Developmental rates during preimplantation and outgrowth formation. Zygotes injected with *mCherry-Trim21* mRNA, *n* = 212; zygotes injected with *mCherry-Trim21* mRNA and anti-GFP antibody, *n* = 245; zygotes injected with *mCherry-Trim21* mRNA and anti-TEAD4 antibody, *n* = 501. Blastocysts tested for outgrowth formation: *n* = 12 after *mCherry-Trim21* mRNA, *n* = 8 after *mCherry-Trim21* mRNA and anti-GFP antibody, *n* = 12 after *mCherry-Trim21* mRNA and anti-TEAD4 antibody. **c.** Developmental rates after blastocyst transfer to uterus and representative pictures of uteri. Postimplantation development of TEAD4-depleted embryos was lower than that embryos injected with *mCherry-Trim21* mRNA or with *mCherry-Trim21* mRNA + anti-GFP antibody. Day E3.5 blastocysts containing *mCherry-Trim21* mRNA, *n* = 30 in three recipients; day E3.5 blastocysts containing *mCherry-Trim21* mRNA and anti-TEAD4 antibody, *n* = 30 in three recipients; day E3.5 blastocysts containing *mCherry-Trim21* mRNA and anti-GFP antibody, *n* = 48 in five recipients. Implantation rate: (fetuses + empty decidua + miscarriages) / transferred embryos. Fetal rate: fetuses / transferred embryos (see Methods). **d.** Raw Affymetrix signal intensities of selected mRNAs in blastocysts, in logarithmic scale. Two pools of 10 blastocysts each were analyzed for each group (*mCherry-Trim21* mRNA, anti-GFP, anti-TEAD4). Size bar, 50 μm. OGDB, Oregon Green dextran beads. Error bars = standard deviations. Statistical significance tested with Student’s t test. n.s., not significant. AU, arbitrary units of Affymetrix hybridization intensity

Functional consequences were assessed by immunofluorescence and implantation assays. At day E3.5, immunofluorescence intensity of TEAD4 was significantly reduced from a level of 26.8 arbitrary units (AU) in control (*mCherry-Trim21* mRNA) to 19 AU in *TEAD4-depleted embryos* (− 29%); TEAD4’s target CDX2 was significantly reduced from 26 AU to 6.8 AU (− 74%) (Fig. 5d). This substantial - albeit incomplete - TEAD4 depletion was sufficient for a strong functional impairment (Fig. 6a): only 22 ± 20% of the embryos formed a blastocyst cavity at day E3.5, in contrast to 82 ± 18% blastocysts of the group injected with anti-GFP antibody. It was reported that blastocyst cavity formation in *Tead4* −/− embryos is promoted by low O_2_ and antioxidants [55]. In our hands, culture under the more physiologic 5% O_2_ did not improve blastocysts formation of TEAD4-depleted embryos (5% O_2_*:* 0*%, n* = 60) nor did culture in medium containing the antioxidant N-acetylcysteine (NAC), which is also an inhibitor of the endoplasmic reticulum stress response (NAC: 20 ± 7% blastocysts formation, *n* = three groups of 10 zygotes each). TEAD4-depleted embryos were strongly impaired at forming outgrowths in vitro (Fig. 6a, b) and implanting in the uterus (Fig. 6c), in contrast to controls injected with *mCherry-Trim21* mRNA and anti-GFP.

Since TEAD4 is a transcription factor, the effect of TRIM21-mediated protein depletion can be assessed via transcriptome analysis, expecting to see that *Tead4* target genes [34] but not other genes are affected in TEAD4-depleted day E3.5 blastocysts. Transcriptome analysis was conducted on three groups of *n* = 10 embryos at day E3.5: 1) *mCherry-Trim21* mRNA; 2) *mCherry-Trim21* mRNA + anti-GFP; and 3) *mCherry-Trim21* mRNA + anti-TEAD4), in duplicate. The raw microarray data are accessible through GEO Series accession number GSE124844. A summary of the processed microarray data is provided as Additional file 2: Table S2. A marked underexpression of trophectoderm (but not pluripotency-associated) marker genes was observed after injection of *mCherry-Trim21* mRNA + anti-TEAD4. Exemplarily, *Cdx2* mRNA level was reduced to 1124 AU from the starting level of 3805 AU (anti-GFP) and 4315 AU (*mCherry-Trim21* mRNA), which corresponds to a 70–74% knockdown (t test, *p* < 0.01; Fig. 6d). Marked reductions were observed also in the level of *Gata3* mRNA (down to 146 AU from the starting level of 326 and 351 AU for anti-GFP and *mCherry-Trim21* mRNA, respectively; − 55%; − 58%) and of the other trophectoderm marker mRNAs (*Krt8, Cdh1, Atp1b1*), except *Eomes* (Fig. 6d). In contrast to these trophectodermal mRNAs, levels of housekeeping mRNAs and pluripotency-associated *Nanog* and *Pou5f1* mRNA were not changed, while levels of *Sox2* were – if anything – slightly increased (Fig. 6d)*.* Overall, counting in the two mRNAs of *Cdx2* and *Gata3*, 280 genes were affected by TEAD4 antibody (t test, *p* < 0.01). We applied also a fold-change threshold. Of the total 280 mRNAs, 36 were changed by more than twofold and 7 by more than fourfold (*Lgals1, Id2, Slc44a4, Krt18, Oas1f, Obox6, Cdx2*). A total of 182 transcripts were affected by GFP antibody (t test, *p* < 0.01), 8 of which were also changed by more than twofold and 2 by more than fourfold (*Mir3079, Dkk1*). By these thresholds, various elements of the endoplasmic reticulum stress response (*Eif2ak3/Perk, Map 3 k5/Ask1, Hspa5/Bip, Ddit3/Chop, Ppp1r15a/Gadd34, Ern1/Ire1, Atf4, Atf6, Xbp1* [56, 57]) were not perturbed by anti-GFP or anti-TEAD4 antibody compared to embryos treated with *mCherry-Trim21* mRNA (*p* values ≥0.11 and fold-changes ≤1.6). Thus, microinjection of antibody against a resident protein, namely TEAD4, has specific consequences, however, there might be also some unspecific effects of the antibody as such, as revealed by the antibody against a target (GFP) not present in the wildtype cell.

**Fig. 7:**
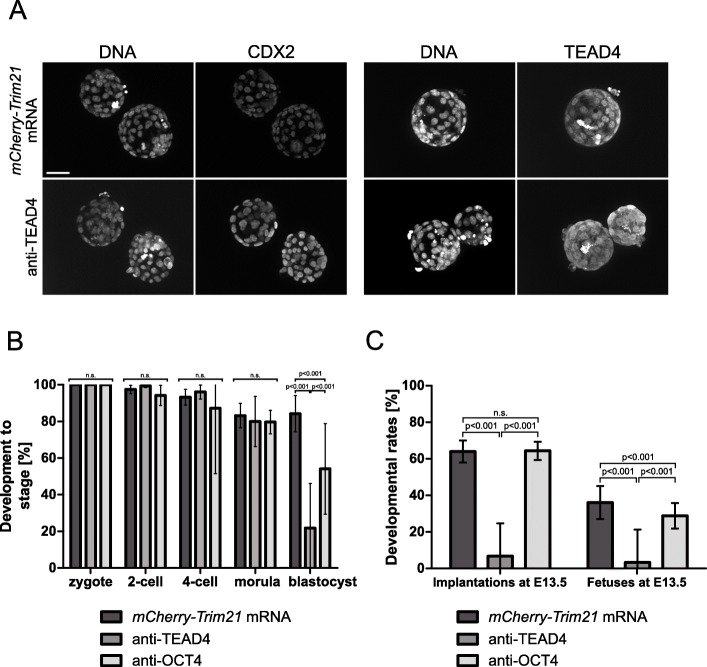
**a** Representative immunofluorescent signals of TEAD4 and CDX2 in TRIM21-only and TEAD4-depleted embryos at day E4.5 (*n* = 8 TEAD-depleted and *n* = 6 TRIM21-only embryos for TEAD4 immunofluorescence; *n* = 10 TEAD-depleted and *n* = 6 TRIM21-only embryos for CDX2 immunofluorescence). DNA stained with YO-PRO-1. Size bar, 50 μm. **b.** Developmental rates during preimplantation. Zygotes injected with *mCherry-Trim21* mRNA, *n* = 212; zygotes injected with *mCherry-Trim21* mRNA and anti-TEAD4 antibody, *n* = 501; zygotes injected with *mCherry-Trim21* mRNA and anti-OCT4 antibody, *n* = 464. **c.** Developmental rates after blastocyst transfer to uterus. Day E3.5 blastocysts containing *mCherry-Trim21* mRNA, *n* = 30 in 3 recipients; E 3.5 blastocysts containing *mCherry-Trim21* mRNA and anti-TEAD4 antibody, *n* = 30 in 3 recipients; E 3.5 blastocysts containing *mCherry-Trim21* mRNA and anti-OCT4 antibody, *n* = 160 in 20 recipients. Implantation rate: (fetuses + empty decidua + miscarriages) / transferred embryos. Fetal rate: fetuses / transferred embryos (see Methods). Statistical significance tested with Student’s t test. n.s., not significant

TRIM21 does not operate on the DNA locus, which continues to transcribe RNA that continues to be translated. Therefore, it is possible that the phenotype observed on day E3.5 may recover later. Indeed on day E4.5, TEAD4-depleted blastocysts had restored CDX2 and TEAD4 expression (Fig. 7a) and upon transfer to uterus developed further at rates indistinguishable from controls. In keeping with the reasoning that TRIM21-mediated protein depletion is effective for 3 days, but not longer, we challenged the product of another gene, namely *Pou5f1 (Oct4),* which is required later in development compared to *Tead4. Pou5f1* −/− embryos form blastocysts, but these have a non-functional inner cell mass [3]*.* When PN2 zygotes were depleted of OCT4 the same way as described for TEAD4, depletion of OCT4 was initiated (Fig. 4d)*,* but blastocyst formation was marginally affected compared with that of zygotes injected with *mCherry-Trim21* mRNA and anti-TEAD4 (Fig. 7b)*.* Following transfer of E3.5 OCT4-depleted blastocysts to uterus, fetal rates were substantial (Fig. 7c)*.* Thus, the effect of TRIM21-protein depletion is confined to 3 days, and appears more suited for low-expressed proteins like TEAD4 (8th percentile of the riBAQ distribution in oocytes) than for high-expressed proteins like OCT4 (65th percentile). These findings prompt us to discuss what the basis of this difference could be, and whether proteins other than TEAD4 and OCT4 may be suited for TRIM21-mediated protein depletion*.*

## Discussion

Developmental biologists have sought for decades to illuminate gene functions in early embryos by testing what happens when a specific gene is disrupted experimentally. While DNA and RNA methods are used commonly to impair gene expression, they are of limited efficacy against the protein products of those genes. Protein methods are therefore desirable to gather a complete picture. Recently the antibody microinjection method [10, 11] has brought a major advance: in addition to the binding of the target (which can mask proteins at the catalytic or interaction sites) it also achieved the subsequent degradation of the antibody-target complex via TRIM21 ubiquitin ligase (TRIM21-mediated protein depletion [21]). Even with this advance, effectiveness of protein depletion to reproduce mutant gene phenotypes in early mouse embryos seems all but granted, because: the effect must last 3–4 days, and microinjection must deliver an amount of reagent that is adequate for the protein amounts present in early embryos, which must be known. Here we have shown that when a specific antibody is available to target the protein of interest and the microinjection method is scaled up so as to deliver maximum amounts of reagent to the mouse ooplasm, substantial (albeit not complete) depletion of gene activity can be accomplished on the protein level for 3 days. Our arena was the PN2 stage wildtype mouse zygote and its acquisition of trophectodermal function mediated by the transcription factor TEAD4. TRIM21-mediated TEAD4 depletion resulted in a phenotype that is entirely consistent with the reported genetic knockout and knock-down of *Tead4*. Our control experiments using antibody against GFP, which is not present in the wildtype cell, and our test for off-target effects using transcriptome analysis, attest to the specificity of the TEAD4 depletion effects. If we prove that TRIM21-mediated protein depletion is effective during development for 3 days, then in principle we should be able to tackle also genes whose product is required earlier than day 3, such as the maternal-effect genes [58, 59].

One conceptual and one technical consideration were fundamental in our study. Firstly, the knowledge of how much of the target protein is present in the oocyte and early embryo is important, so as not to operate blindly when microinjecting the antibody. If the moles of antibody were much less than the moles of target protein, then TRIM21-mediated protein depletion would have no notable effect, regardless of any other consideration. For these reasons, quantitative knowledge about the oocyte proteins is essential. In our case, this knowledge was achieved via mass-spectrometric analysis, revealing the mouse developmental proteome to a depth of over 7000 proteins, which exceeds any other mouse dataset published to date, even the two largest [23, 24]. Probably we have not yet reached saturation in our data, since of the cohesin complex we missed one (REC8) of the 4 core members, for example. This is a shared limitation of all proteomic studies of mouse oocytes and early embryos to date. Our tests ascribe the proteins detected in mouse oocytes and early embryos with the tentative unit of substance of subpicomoles (range from 4.0E-09 to 7.7E-02 picomoles) – among these proteins also TEAD4, toward which we directed our endeavors. It may be noted that TEAD4 was detected in the low end of the protein abundance range, at a time prior to when the embryonic locus starts to be transcribed at the 2-cell stage [2], consistent with several other maternal proteins that are present in embryos in spite of the fact that mRNA is not detected [8]. This quantitative knowledge allows to scale comparable amounts of antibody, provided these amounts are also technically deliverable via microinjection in the zygote.

The second consideration was about achieving a maximum supply of reagent (TRIM21 coding mRNA, and antibody) to the ooplasm, in order to have a match for the endogenous proteins (4.0E-09 to 7.7E-02 tentative picomoles). The mouse oocyte is notoriously fragile during microinjection, and the mRNA amount was the highest we could possibly inject, before the concentration started to become toxic for the mouse embryo. The picomoles of antibody were also the highest possible, and cover all but the 98th and 99th percentile of the oocyte proteome distribution. To deliver these amounts of reagent, an unprecedented large volume of approximately 100 picoliters was injected into a zygote that has a volume of 220 picoliters. Although Zebrafish oocytes were successfully injected with 20 times the volume and 10 times the protein amount we injected into mouse oocytes [25], it should be noted that Zebrafish oocytes are also 300 times larger [22]. It appears that the cell volume regulation abilities of the mouse ooplasm [28] are astounding, such that it does not lose developmental potential to the injection of almost one half of its volume. In contrast to the PN2 zygote, the MII oocyte did not tolerate injection of such a large volume, the reason why we worked with PN2. This is a limitation of our study, because processes that are already in train in PN2 zygotes may be more difficult to disrupt, compared to processes that have yet to start in MII oocytes. The reason why *mCherry-Trim21* mRNA and TRIM21 protein exert some form of developmental toxicity above the concentration of 2.5E-05 picomoles and 9.6E-04 picomoles, respectively, is not clear. This toxic effect could simply be related to a plethora of issues that may be caused by the non-physiological influx of large amounts of substance, or be related specifically to additional functions of TRIM21, which include the induction of autophagy through IFN-γ [60] and the innate defense response to virus [61]. It is also conceivable that by overloading the proteasome with TRIM21-antibody-antigen complex that does not normally exist in the cell, the physiologic process of removal of other proteins is impaired (e.g. autophagy), causing problems to the embryo. Our transcriptomic data do not corroborate these explanations (see below).

Under the provision of 1) a maximum amount of reagents injected, and 2) a valid antibody, the effects of TRIM21-mediated protein depletion persisted for enough time in which to phenocopy the loss of *Tead4* gene function in vitro. Blastocyst and outgrowth formation were strongly impaired in TEAD4-depleted embryos, unlike the GFP control. These data do not rule out non-specific effects of the antibody treatment, however. Since TEAD4 is a transcription factor, transcriptome analysis can reveal if only the target genes of TEAD4 or also other genes are affected, and how many. On the transcriptional level, the effect was a reduced expression of TEAD4 target genes *Cdx2* and *Gata3* (as well as other trophectodermal genes). Only 6 other genes (*Lgals1, Id2, Slc44a4, Krt18, Oas1f, Obox6)* were more differently expressed than *Cdx2* in the comparison between TEAD4-depleted and *mCherry-Trim21* embryos. On the protein level, CDX2 was synthesized in TEAD4-depleted embryos, as logically expected from the above transcriptomic data and consistent with previous reports that documented reduced (but not absent) CDX2 expression in *Tead4*^−/−^ embryos [1, 33, 55]. In spite of the incomplete effect, the functional consequence was a highly compromised trophectodermal function [4], to the point that the E3.5 blastocysts were unable to form outgrowths in vitro. As such, the protein depletion phenotype was consistent with that of the genetically null *Tead4*−/− zygotic knockout originally described [2] and even more severe than *Cdh1 (ECadherin)* −/− zygotic knockout embryos (which were able to form trophectodermal outgrowth, albeit less extended than in control embryos [62]). Thus, targeting the downstream product produces a more severe phenotype, as noted previously for RNA interference compared to genetic null mutations [6]. When tested in vivo by transfer to the uterus, TEAD4-depleted embryos again did not implant when transferred at E3.5, while they succeeded when transferred at E4.5. In order to make sense of this discrepancy we note that the O_2_ tension is lower in the uterus, and that the *Tead4* −/− phenotype might be contingent on the O_2_ tension, having been proposed that TEAD4 operates in mitochondria to protect the cell from reactive oxygen species [55, 63]*.* Unfortunately a comparison of these studies with our study does not contribute to the discussion, because our TEAD4-depleted embryos were cultured without oil overlay from the 1-cell stage, whereas the rescued ones were cultured with oil overlay in the presence of NAC from the 2- or 8-cell stage [55]*.* Regardless, in our study, TEAD4-depleted embryos were equally impaired under 20% or 5% *O*_*2*_ or use of antioxidant NAC, suggesting that the embryos of our study experienced modest cellular stress*.* This view is supported by the marginal change observed in transcripts associated with endoplasmic reticulum stress [56, 57], thereby speaking for a general validity of the TEAD4 data, although cellular stress depends also on environmental factors and operators’ skills that are difficult to reproduce exactly in different laboratories.

The reason why *Cdx2* and *Gata3* were still expressed in spite of the large excess of TEAD4 antibody (6.7E-04 picomoles) compared to its target protein (6.3E-07 picomoles) is unclear, but we showed it does not lie in the premature exhaustion of microinjected reagents. We should consider that not even in the *Tead4*−/− zygotic knockout the expression of all TEAD4 target genes was completely silenced: *Eomes* continued to be expressed, for example [2]. Another possibility is that target proteins are not entirely accessible to TRIM21 when located in some subcellular compartments e.g. nucleus or cortex. Furthermore, the binding properties of the antibody play a role. When the target protein concentration is below the chemical dissociation constant (K_d_) of the antibody [30], binding is under 50%, resulting in incomplete depletion from the outset. When the target protein concentration lies above the K_d_, binding can be complete but this is only a temporary state, since the concentration becomes progressively reduced by TRIM21 reaction. Similar considerations also apply to the binding of TRIM21 to the antibody. The problem of partial binding will arise, at the latest, when the target protein concentration has fallen below the K_d_ of the antibody, or the concentration of the antibody-antigen complex has fallen below the K_d_ of TRIM21.

## Conclusions

TRIM21-mediated protein depletion promises to induce null-like developmental phenotypes without using genetic tools, and to tackle previously synthesized proteins, which accumulated in the oocyte even before the gene locus was removed (in knockout models) or the mRNA was inhibited (in siRNA/morpholino experiments). Our fact-check with TEAD4 in the canonical mammalian model, the mouse, demonstrates success for 3 days, in conjunction with some prerequisites, requirements and limitations. Key prerequisite is a quantitative knowledge of the oocyte’s and embryo’s proteome, so as to estimate the amount of protein the antibody has to react with. Key requirement is the ability to supply that amount via volume and concentration of a single microinjection. However, getting large amounts of pure, high-affinity antibodies into the oocyte or zygote is not only challenging, but also quite costly. Key limitation of our method is that binding capacities decline as development progresses (antibody is used up and cannot bind 100% of the target protein anyway, given the chemical equilibrium law), while the endogenous gene source is still active, whereby protein function will be restored sooner or later. This means that the system is leaky and the dream of completely removing any gene product might not be accomplishable, except for some carefully selected proteins. We demonstrated TEAD4 depletion for 3 days starting from the zygote, sufficient to phenocopy the lethal *Tead4* null mutation, but perhaps insufficient for other gene products that are later acting, or more abundant, or shielded in a subcellular compartment. This temporal profile suggests that the method may actually be more suited for oocyte deposits of gene products that are downregulated in the embryo, than for embryonic genes that are upregulated. This seems attractive, for example, in order to create a molecular *tabula rasa* in oocytes prior to fertilization or somatic cell nuclear transfer, so as to dissect the function of maternal-effect or reprogramming genes, respectively. Although we have used PN2 zygotes to prove the method, MII oocytes will be the real test bench in future studies. They do not tolerate the microinjection of as much volume as PN2 zygotes, but this is only a technical problem and there are pharmacological means to inhibit the endoplasmic reticulum stress response [57]. In conclusion, lessons from biology (continued gene locus activity) and chemistry (K_d_ of the antibody) indicate that TRIM21-mediated protein depletion cannot remove 100% of the target protein, no matter how large the amount of antibody injected. Yet this partial depletion can disrupt a gene’s product and thereby reveal the gene function.

## Methods

### Compliance with regulations on research animals

All mice were maintained in individually ventilated cages in the animal facility of the MPI Münster, with a controlled temperature of 22 °C, a 14/10 h light/dark photoperiod and free access to water and food (Harlan Teklad 2020SX). Mice were used for experiments according to the license issued by the Landesamt für Natur, Umwelt und Verbraucherschutz of the State of North Rhine-Westphalia, Germany (license number 81–02.04.2017.A432), in accordance with the procedures laid down in the European Directive 2010/63/EU.

### Collection of mouse zygotes and in vitro embryo culture

Six- to eight-week-old B6C3F1 females were primed with 10 I.U. each pregnant mare serum gonadotropin (PMSG; Pregmagon, IDT) and human chorionic gonadotropin (hCG; Ovogest, Intergonan) injected intraperitoneally 48 h apart. MII oocytes were collected from the oviducts. To collect zygotes, primed females were mated to CD1 stud males. On the morning of the vaginal plug, the cumulus-oocyte complexes were recovered from the oviducts at 9 am, dissociated in hyaluronidase (50 I.U./mL in Hepes-buffered Chatot-Ziomek-Bavister (CZB) medium) and cultured in 500 μL of potassium simplex optimization medium containing aminoacids KSOM (aa) in a four-well Nunc plate without oil overlay, at 37 °C under 6% CO_2_ in air. Small scale experiments were conducted also under 5% O_2_ atmosphere (gas mixture 5/5/90) and in the presence of 0.5 mM N-acetylcysteine (NAC) in medium [55]. KSOM (aa) was synthesized from individual components and included 0.5X EAA, 0.5X NEAA and 0.5X glutamine according to recipe [64]. Developmental stages were collected from the plate at the appropriate time points (MII oocyte, 16 h post hCG (hphCG); 1-cell stage, 16 hphCG; 2-cell stage, 43 hphCG; 4-cell stage: 53 hphCG; 8-cell stage, 62 hphCG; morula: 72 hphCG; blastocyst, 92 hphCG).

### Proteome analysis of oocytes, zygotes and preimplantation embryos

For the aims of this study we made use of already existing datasets that we had generated previously [23, 65] using the ‘stable isotope labeling by/with amino acids in cell culture’ (SILAC) pipeline [66]. The original samples had been spiked with a standard prepared from F9 embryonal carcinoma (EC) cells [67, 68]. F9 EC cells build tumors (teratomata) that are considered as caricatures of embryogenesis, because they can differentiate into almost every tissue [69], therefore F9 EC cells afford an ample coverage of the proteins expressed in early embryos. Spiked-in samples had been prepared using either the FASP protocol (exp0313, exp0335) or offline high-pH reversed-phase chromatography of tryptic peptides with concatenated fractionation (exp0616, exp0672). These datasets were supplemented with further experiments unpublished so far, which followed the same sample preparation procedures as above (FASP: exp0471; high-pH RP-chromatography: exp0746; exp0812; exp0860). Briefly, oocytes and embryos had been deprived of the zona pellucida by pipetting in warm acidic Tyrode solution for 30–60 s and then rinsing in protein-free Hepes-buffered CZB medium (BSA replaced through *polyvinylpyrrolidone* 40 kDa). To produce each individual sample 200–300 oocytes or embryos were lysed in 15–20 μl of SDS lysis buffer (4% SDS, 50 mM HEPES pH 7.5) and stored at − 80 °C until further processing. Each oocyte or embryo lysate was supplemented with an equal amount of protein lysate from isotopically labeled (Lys8 and Arg10) F9 EC cells as SILAC spike-in standard (> 98% labeling efficiency). These 1:1 mixtures were then either processed according to the FASP procedure [70] or digested with Lysyl endopeptidase and trypsin, desalted, and fractionated by offline high-pH reversed-phase chromatography. Last, all samples were analyzed by liquid chromatography-mass spectrometry (LC-MS/MS), either on a LTQ Orbitrap Velos or a Q-Exactive mass spectrometer as described in our previous work [23, 65]. Raw mass spectrometry data were deposited to the PRIDE repository via the ProteomeXchange Consortium (http://proteomecentral.proteomexchange.org) [39, 40] with the dataset identifier PXD012613. Raw data were processed for identification and quantification by MaxQuant Software (version 1.6.2.10, [71]), considering only the “light” versions of the proteins, with the options ‘requantify’ and ‘iBAQ’ enabled. iBAQ stands for ‘intensity-based absolute quantification’. MaxQuant enables high peptide identification rates, individualized p.p.b.-range mass accuracies and proteome-wide protein quantification). For identification, the search was performed against the UniProt mouse database (release date 12/2015) concatenated with reversed sequence versions of all entries and supplemented with common contaminants. Parameters defined for the search were trypsin as digesting enzyme, allowing two missed cleavages, a minimum length of seven amino acids, carbamidomethylation at cysteine residues as fixed modification, oxidation at methionine, and protein N-terminal acetylation as variable modifications. The maximum allowed mass deviation was 20 ppm for the MS and 0.5 Da for the MS/MS scans. Protein groups were regarded as identified with a false discovery rate (FDR) set to 1% for all peptide and protein identifications; in addition, at least two matching peptides were required and at least one of these peptides had to be unique to the protein group. Briefly, a protein group is defined as all proteins that are identified by the same set of peptides, which are not included (all together) in any other protein group. In this study we focused on the iBAQ values of the ‘light’ peptide versions only (= peptides derived from oocyte proteins but not from the F9 spike-in standard). The iBAQ algorithm allows to calculate the abundance of proteins within one sample by summing all peptide peak intensities detected for a given protein and normalizing it by the number of theoretically observable tryptic peptides for this protein. Thus, a mass-related measure (intensity) is transformed into a measure that is proportional to molar amounts (iBAQ). iBAQ values for each protein were then divided by the sum of all iBAQ values for a given experiment to determine the molar fractional content of each protein P (riBAQ_P_) in a sample according to the formula adapted from (37):

$$ {riBAQ}_P=\frac{riBAQ_P}{\sum \limits_1^n iBAQ} $$

Last, the riBAQ values were averaged within each stage.

### *mCherry-Trim21* mRNA preparation for TRIM21-mediated protein depletion

For in vitro transcription, plasmid pGEMHE-mCherry-mTrim21 (Addgene plasmid # 105522, was a gift by Melina Schuh) was linearized with SwaI (ThermoFisher, cat. no. FD1244). Capped mRNA was synthesized with T7 polymerase (Ambion mMessage mMachine T7 kit) according to manufacturer’s instructions. Obtained *mCherry-Trim21* mRNA was purified with Quick-RNA MicroPrep (Zymo Research, cat. no. R1051) and preserved in MilliQ water at − 80 °C.

### Protein preparations for TRIM21-mediated protein depletion

To draw a dose-response curve for increasing concentrations of TRIM21 protein, recombinant mouse TRIM21 protein (Biomatik, cat. no. RPC23188) was used. For target protein depletion, antibodies were anti-OCT4 (Santa Cruz SC9081, rabbit polyclonal), anti-PDIA3 (Abcam ab228789, rabbit polyclonal), and anti-TEAD4 (Abcam ab58310, mouse monoclonal, ChIP grade). According to manufacturer anti-TEAD4 antibody is supplied in PBS. For control experiments an anti-GFP antibody was used (ThermoFisher GF28R, mouse monoclonal). TRIM21 protein and antibodies were concentrated at 4 °C using Amicon Ultra-0.5 30 or 100 KDa centrifugal filter devices (Merck Millipore, cat. no. UFC30/ UFC100) to remove salts and preservatives (e.g. sodium azide) and stabilizers (e.g. albumin), and to replace the buffer with water.

### Microinjection of mRNA and antibody in zygotes and blastomeres

In order to stringently test the antibody prior to full-scale experiments, its effect was tested in 2-cell embros using the non-injected blastomere as internal control. Pronuclear-stage oocytes (PN2 zygotes) were injected with a mixture of mRNA and dextran beads fluorescently labeled with Oregon Green (70 kDa; ThermoFisher cat. no. D7173) at the final concentration of 0.2 mg/mL and 0.025 mg/mL, respectively, dissolved in MilliQ water. On the following day, 2-cell embryos were injected in one blastomere with a mixture of antibody and dextran beads at the final concentration of 1.0 mg/mL and 0,025 mg/mL, respectively. Microinjection was conducted on the stage of a Nikon TE2000U microscope fitted with a piezo drill (PrimeTech), using a blunt-end glass needle (inner diameter 4–5 μm, outer diameter 6–7 μm) filled with 2–3 μl mercury at the tip. Volumes were pressure-injected into the zygote or blastomere using a Gilmont GS-1200 μm syringe operated manually. During the microinjection, cells were kept in a 200–300 μl drop of Hepes-buffered CZB medium [72] on a glass-bottomed (Nomarsky optics) dish at a room temperature of 28 °C. After microinjection, zygotes or embryos were allowed to recover in the drop for 5–10 min, before returning them to KSOM (aa) medium. For full-scale experiments, microinjections were performed as above, except that mRNA and antibody were injected sequentially in pronuclear-stage oocytes (PN2 zygotes).

### Fluorescence intensity measurement of mCherry

To measure the fluorescence intensity of mCherry (Figs. 3c, 4b-f, 5b, c), images were captured using a 10X objective, Nikon ACT-2 U camera system and a fixed exposure of 1 s. Regions of interest were drawn around the embryos using Image-J, and the closed line regions were measured with “measure” tool. Only the net average intensity, which is obtained by subtracting the total average intensity with background intensity, was used for final statistics.

### TaqMan analysis of *Trim21* mRNA

Total RNA was isolated using Quick-RNA™ MicroPrep (Zymo Research) following the manufacturer’s instructions and was reverse-transcribed on a GeneAmp® PCR System 9700 (Applied Biosystems). Real-time quantitative PCR reactions were performed on a 7900 HT FAST Realtime PCR System (Applied Biosystems). The cDNA equivalent of ~ 5 embryos per stage was used for each target gene. PrimeTime®Predesigned qPCR Assay (6-FAM/ZEN/IBFQ) from Integrated DNA Technologies was used. Assay IDs: Trim21: Mm.PT.5812570300 and β-Actin: Mm.PT.39a.22214843.g. All samples were processed as technical duplicates. Data were analyzed with the ΔΔCt method [73] using the Applied Biosystems RQ Manager (Version 1.2.2) and Microsoft Excel. ∆∆Ct = ∆ (Ct Trim21 - Ct β-Actin of mCherry-Trim21 injected embryos) – ∆ (Ct Trim21 - Ct β-Actin of non-injected wt embryos). Ct: cycle threshold.

### Transcriptome analysis of blastocysts

On day 4 after microinjection, two replicates each of three groups were created: Trim21 mRNA and dextran beads (named ‘group 4’), or Trim21 mRNA, dextran beads and anti-GFP antibody (named ‘group 5’), or Trim21 mRNA, dextran beads and anti-TEAD4 antibody (named ‘group 6’). Total RNA was extracted using the ZR RNA Microprep Kit (Zymo Research Corporation, Irvine, USA) without the DNase digestion step. Gene expression profiling was performed using Affymetrix GeneChip® Mouse Transcriptome Array 1.0 (Affymetrix United Kingdom Ltd., High Wycombe, UK) containing < 214000 transcripts. The fragmented and biotinylated DNA targets were prepared according to the standard Affymetrix WT Pico Reagent Kit protocol (Affymetrix GeneChip® WT Pico Reagent Kit) using 11 amplification cycles from the total RNA starting material available. GeneChips were hybridized, washed and stained in the Affymetrix Fluidics Station 450, according to the standard GeneChip Expression Wash, Stain and Scan protocol (Affymetrix GeneChip Wash, Stain and Scan Kit). Hybridization took place at 45 °C for 16 h. The GeneChips were scanned using the Affymetrix 3000 7G scanner. The Affymetrix Expression Console and Transcriptome Analysis Console was used for microarray data analysis. The robust multiarray averaging method was applied for background correction, normalization and probe summarization. Gene expression differences were determined by applying an analysis of variance.

### Immunofluorescence analysis of GFP antibody and TEAD4/CDX2 expression

Embryo were analyzed by performing an immunostaining followed by confocal microscopy imaging. The following primary antibodies were applied to the specimens overnight at 4 °C: anti-CDX2 mouse IgG1 (Emergo Europe, The Hague, Netherlands, cat. no. CDX2–88) and anti-TEAD4 (Abcam 58310) in dilutions of 1:200 and 1:100, respectively. For GFP antibody stability experiment, eGFP polyclonal antibody (ThermoFisher, cat.no. CAB4211) was injected into zygotes and embryos were fixed after 0 h, 24 h, 48 h, 72 h and 96 h. Appropriate Alexa Fluor-tagged secondary antibodies (Invitrogen) were matched to the primaries and incubated for 1–2 h at room temperature. DNA counterstaining was performed with YO-PRO-1 (1 micromolar). For imaging, embryos were placed in 5 μl drops of PBS on a 50-mm thin-bottom plastic dish (Greiner Bio-One, Lumox hydrophilic dish; Frickenhausen, Germany) and overlaid with mineral oil (M8410 Sigma). Images were captured on the stage of an inverted microscope (Eclipse 2000-U; Nikon, Düsseldorf, Germany) fitted with a spinning disk confocal unit (Ultra View RS3; Perkin-Elmer LAS, Jügesheim, Germany). A Nikon Plan Fluor 40X oil immersion lens (NA 1.30) was used. Twenty optical sections per embryo were captured using a Hamamatsu ORCA ER digital camera (Hamamatsu Photonics KK, Japan). Maximum projections were analyzed with ImageJ Version 1.46j.

### Outgrowth formation by blastocysts

Zona-free Blastocysts were transferred onto a feeder layer of γ-ray-inactivated mouse embryonic fibroblasts (C3H background) grown to confluence in 12-well plates (flat bottom) previously. The culture medium consisted of high-glucose DMEM (Gibco) with 15% fetal bovine serum (BioWest, Nuaillé, France), glutamine and penicillin/streptomycin (Gibco), non-essential amino acids (PAA Laboratories, Pasching, Austria), mercaptoethanol 0.1 mM (Gibco). Within 4 days of the transfer onto fibroblasts, healthy blastocysts would attach to the feeder layer and form trophoblastic outgrowths.

### Embryo transfer and post-implantation development

Groups of 10 blastocysts were transferred surgically to one uterine horn of pseudopregnant CD1 recipients that had received the copulation plug from vasectomized CD1 males 2 days prior to the embryo transfer. Prior to surgery, CD1 foster mothers were anesthetized with Ketamin (80 mg/kg body weight)/Xylazin (16 mg/kg)/Tramadol (15 mg/kg) in PBS, delivered intraperitoneally. Pregnancies from embryos with mCherry-TRIM21 alone or with anti-GFP were evaluated by C-section just prior to term (embryonic day (E) 18.5); pregnancies from embryos with mCherry-TRIM21 and anti-TEAD4 were evaluated on E13.5 so as to be in a better position to assess early developmental losses. Fetal rate was calculated as the number of fetuses of regular size for gestational age at E13.5, divided by the number of embryos that had been transferred. Implantation rate was calculated as the total number of fetuses, of empty decidua and of miscarriages, divided by the number of embryos that had been transferred.

### Statistical analysis of developmental rates, image data and gene expression data

Blastocyst rates and fluorescence intensities were analyzed by two-tailed Student’s *t* tests using R and the statistical program JMP v.13 (SAS). Microarray data analysis was performed in-house using the output of the Affymetrix Expression Console and Transcriptome Analysis Console, exported in Excel format and imported in JMP v.13. Likewise, iBAQ data were analyzed using Excel and JMP v.13. Graphs were created with GraphPad PRISM software.

## Supplementary information

**Additional file 1: Table 1.** Summary of the processed riBAQ data.

**Additional file 2: Table 2.** Summary of the processed Affymetrix data.

## Data Availability

The mass spectrometry proteomics data have been deposited to the ProteomeXchange Consortium via the PRIDE partner repository [39, 40] with the data set identifier PXD012613. The microarray data generated and analyzed in this article have been deposited in the NCBI’s Gene Expression Omnibus and are accessible through GEO Series accession number GSE124844. Summary tables of PXD012613 and GSE124844 are provided in supplementary material.

## Abbreviations

AB: Antibody
AU: Arbitrary units
CZB: Chatot Ziomek Bavister (medium)
EC: Embryonal carcinoma (cell)
GFP: Green fluorescent protein
hCG: human chorionic gonadotropin
I.U.: International units
iBAQ: intensity Based Absolute Quantification
K_d_: Dissociation constant
KSOM (aa): Potassium simplex optimization medium containing aminoacids
LC-MS/MS: Liquid chromatography-mass spectrometry
MII: Metaphase II (oocyte)
OGDB: Oregon green-labeled dextran beads
PMSG: Pregnant mare serum gonadotropin
PN2: Pronuclear-stage two (oocyte)
riBAQ: relative iBAQ
SCMC: Subcortical maternal complex
SILAC: Stable isotope labeling by/with amino acids in cell culture
TEAD4: TEA Domain Transcription Factor 4
TRIM21: **TRI**partite **M**otif-containing **21** (protein)
